# Recombinant hCG versus dual triggering for final oocyte maturation in ICSI cycles among poor responders with endometriosis

**DOI:** 10.1530/RAF-25-0215

**Published:** 2026-10-20

**Authors:** Fatemeh Davari Tanha, Mahbod Ebrahimi, Zahra Kaveh, Elham Feizabad, Shirin Zarrinjooie, Hakimeh Roshani, Shima Rahimi, Elham Rashidi

**Affiliations:** ^1^ Department of Obstetrics and Gynecology, School of Medicine, Yas Hospital, Tehran University of Medical Sciences, Tehran, Iran; ^2^ School of Medicine, Tehran University of Medical Sciences, Tehran, Iran; ^3^ Department of Community Medicine, School of Medicine, Tehran University of Medical Sciences, Tehran, Iran; ^4^ Prehospital and Hospital Emergency Research Center, Tehran University of Medical Sciences, Tehran, Iran

**Keywords:** recombinant HCG, dual trigger, GnRH agonist, ICSI, endometriosis

## Abstract

**Abstract:**

This double-blind randomized clinical trial compared recombinant human chorionic gonadotropin (rhCG) alone with dual triggering (gonadotropin-releasing hormone agonist plus rhCG) for final oocyte maturation in women with endometriosis and poor ovarian response (Bologna criteria) undergoing intracytoplasmic sperm injection. A total of 152 eligible women were randomized (1:1) to receive either rhCG (250 μg) plus placebo (*n* = 76) or rhCG (250 μg) plus GnRH agonist (0.2 mg; *n* = 76). Ultimately, 128 participants completed the protocol through embryo transfer (per-protocol cohort: *n* = 65 rhCG, *n* = 63 dual trigger). The primary outcome was the number of retrieved metaphase II (MII) oocytes; secondary outcomes included embryo quality and pregnancy rates. The dual-trigger group yielded significantly more MII oocytes (6.53 ± 4.61 vs 4.14 ± 2.91; effect size (ES) = 0.63, 95% CI: 0.30–0.95; *P* < 0.001) and Grade A embryos (0.66 ± 0.82 vs 0.37 ± 0.62; ES = 0.39, 95% CI: 0.05–0.73; *P* = 0.023). Per-protocol chemical pregnancy (38.1 vs 15.4%; relative risk of success (RR) = 2.48, 95% CI: 1.29–4.75; *P* = 0.004) and clinical pregnancy rates (38.1 vs 10.8%; RR = 3.54, 95% CI: 1.64–7.62; *P* < 0.001) were significantly higher following dual triggering, with consistent findings in intention-to-treat analyses. Dual triggering significantly enhances oocyte maturity, embryo quality, and clinical pregnancy outcomes compared with rhCG monotherapy in poor responders with endometriosis; larger multicenter trials are warranted to corroborate these findings.

**Lay summary:**

Women with endometriosis often face difficulty becoming pregnant, especially when their ovaries produce only a small number of eggs. In assisted fertility treatments, hormones are used to help eggs mature before they are collected. One common method uses a hormone called hCG, whereas another approach, known as dual triggering, combines hCG with an additional hormone to better mimic the body’s natural process. In this study, we compared these two methods in 152 women with endometriosis and poor ovarian response undergoing fertility treatment. We found that women who received the two-hormone method produced more fully mature eggs and more good-quality embryos, and had higher pregnancy rates than those who received hCG alone. These findings suggest that using both hormones may offer a better chance of success for some women with endometriosis undergoing fertility treatment and could help doctors tailor treatment to individual patient needs.

## Introduction

Infertility affects more than 10% of couples of reproductive age worldwide (Haas *et al.* 2019). Among the diverse etiologies, endometriosis – a chronic, estrogen-dependent inflammatory disease – is frequently implicated in reproductive failure. Current literature suggests that patients with endometriosis exhibit significantly impaired folliculogenesis (Kuivasaari *et al.* 2005). This endometriosis-associated infertility represents a multifactorial clinical challenge, driven by a cascade of systemic and local dysfunctions that compromise oocyte developmental competence. At the systemic level, dysregulation of the hypothalamic–pituitary–ovarian (HPO) axis disrupts the precision of gonadotropin surges, thereby impairing the endocrine cues essential for follicular priming. Concurrently, the ovarian microenvironment is characterized by defective steroidogenesis and persistent chronic inflammation, which inherently limit follicular responsiveness to ovulatory triggers. This pathological milieu is further exacerbated by oxidative stress – manifested by elevated reactive oxygen species – which jeopardizes critical cellular processes, including spindle assembly and mitochondrial bioenergetics. Collectively, these multilevel disruptions culminate in a profound asynchrony between nuclear and cytoplasmic maturation (Stilley *et al.* 2012). This fundamental failure may explain the clinical observation of significantly higher rates of immature (metaphase I (MI) and germinal vesicle (GV)) oocytes in assisted reproductive technology (ART) cycles, a finding that persists independently of the mechanical impact of endometriomas (Hamdan 2016). In this context, dual triggering – combining a GnRH agonist (GnRH-a) with human chorionic gonadotropin (hCG) – may more closely replicate physiological gonadotropin surges, enhance follicular responsiveness, and improve oocyte maturation outcomes in patients with endometriosis.

Controlled ovarian hyperstimulation (COH) remains a cornerstone of ART, aiming to promote multifollicular development to maximize oocyte yield and the subsequent likelihood of generating viable embryos (Schieve *et al.* 2010). However, endometriosis profoundly impacts ART success. A landmark meta-analysis by Barnhart *et al.*, encompassing 22 studies, demonstrated that patients with endometriosis had lower pregnancy rates, reduced fertilization and implantation rates, and a diminished number of retrieved oocytes compared with controls with tubal-factor infertility (Barnhart *et al.* 2000). Furthermore, Sanchez *et al.* provided evidence suggesting that impaired oocyte quality, rather than compromised endometrial receptivity, primarily drives suboptimal reproductive outcomes in this population. Proposed mechanisms for this decline in quality include granulosa cell dysfunction, impaired steroidogenesis, alterations in the follicular fluid microenvironment, and structural oocyte abnormalities involving spindle defects and mitochondrial dysfunction (Sanchez *et al.* 2017).

Ovarian hyperstimulation for ART involves three main elements: promoting multifollicular growth with gonadotropins, preventing the LH surge to avoid premature ovulation, and restoring LH function to trigger oocyte maturation. The chosen triggering method significantly impacts the safety and success of ART procedures. In a retrospective study, 50 women in each arm underwent ovulation triggering using a flexible GnRH-a protocol. Women at risk of ovarian hyperstimulation syndrome (OHSS) were excluded. The authors concluded that dual triggering with this flexible protocol for oocyte maturation did not improve intracytoplasmic sperm injection (ICSI) outcomes (Shakerian *et al.* 2021). GnRH-a stimulates a surge of both FSH and LH, mimicking the body’s natural process. hCG induces only LH activity without FSH involvement. The dual-trigger approach (GnRH-a plus hCG) enhances final oocyte maturation by promoting cumulus cell expansion, increasing LH receptor activity, and improving oocyte competence. These effects contribute to more complete oocyte maturation, higher fertilization rates, and improved embryo quality.

Accumulating evidence from studies published between 2022 and 2025 suggests that a dual-trigger strategy confers significant clinical benefits in *in vitro* fertilization (IVF)/ICSI cycles, particularly within fresh embryo transfer frameworks (Ren *et al.* 2022, Hsia *et al.* 2023, Chen *et al.* 2024, Riobó *et al.* 2025, Somer *et al.* 2025). Specifically, a 2025 meta-analysis restricted to randomized controlled trials (RCTs) demonstrated superior live birth rates (LBRs) and clinical pregnancy rates (CPRs), although no consistent improvement was observed in oocyte yield or maturity with dual-trigger protocols; however, these advantages were less pronounced or inconsistent in frozen embryo transfer (FET) cycles (Beebeejaun *et al.* 2025). While broader pooled analyses encompassing both normal and poor responders indicate improved oocyte maturation and reproductive outcomes, these findings remain highly variable because of heterogeneity in study designs, patient selection, and controlled ovarian stimulation protocols. For patients with poor ovarian response (POR), pooled estimates trend toward improved CPRs and LBRs; however, reliance on observational studies and divergent diagnostic classifications limits the robustness of this evidence (Riobó *et al.* 2025). Although network meta-analyses (NMAs) corroborate the utility of dual triggers in selected patient subsets, they simultaneously highlight a critical requirement for large-scale, standardized RCTs to validate these outcomes across diverse clinical populations (Beebeejaun *et al.* 2025).

For instance, in a single-center, randomized, double-blind controlled trial including 155 normal-responder patients, participants were randomly assigned to receive either hCG alone or a dual trigger for final oocyte maturation. The use of dual triggering for final follicular maturation resulted in a higher number of retrieved oocytes, a greater number of mature oocytes, and an increased number of blastocysts (both total and top quality) compared with triggering with hCG alone (Haas *et al.* 2020). In a review of 1,285 poor–responder cycles, Mutlu *et al.* reported that dual triggering significantly increased the number of MII oocytes, fertilization rates, CPRs, and LBRs compared with hCG alone (Mutlu *et al.* 2022). In contrast, Keskin *et al.* compared cycle outcomes of dual triggering versus hCG alone in normoresponders (NRs) and in patients classified according to the Patient-Oriented Strategies Encompassing Individualized Oocyte Number (POSEIDON) criteria in a randomized controlled trial. A total of 225 participants were allocated to the study groups, with the intervention arm receiving a dual trigger and the control arm receiving hCG alone. They reported a lower LBR in the dual-trigger group (19.2%) than in the hCG group (39.2%), while similar LBR per embryo transfer (ET) was observed between the NR dual-trigger and hCG-only groups. However, further analysis suggested that maternal age and the number of embryos transferred influenced these differences, indicating potential confounding factors (Keskin *et al.* 2023).

Despite growing evidence supporting dual triggering across various ART populations, its efficacy remains debated. Notably, the majority of existing research has focused on either general poor responders or normal responders, leaving a significant knowledge gap regarding patients who present with the dual challenge of endometriosis and POR. Given that endometriosis independently impairs oocyte quality through distinct inflammatory and oxidative pathways, the response to dual triggering in this specific subgroup may differ from that in other IVF populations. To date, few randomized controlled trials have specifically evaluated whether dual triggering can overcome the unique maturation defects associated with endometriosis-related POR. Therefore, this study aimed to evaluate the number of metaphase II (MII) oocytes, embryo grade, and pregnancy outcomes following ovulation triggering with recombinant hCG alone compared with dual triggering in ICSI cycles among women with endometriosis and low ovarian reserve. We hypothesized that dual triggering with a GnRH agonist plus recombinant hCG would increase the number of mature (MII) oocytes and improve clinical pregnancy outcomes compared with recombinant hCG alone in this specific population.

## Materials and methods

This double-blind randomized controlled trial was conducted at a university-based hospital among women with endometriosis and POR undergoing ICSI. The primary outcome was the number of MII oocytes retrieved per cycle. Secondary outcomes included embryo quality, chemical pregnancy rate, and clinical pregnancy rate. Embryo morphology was assessed by experienced embryologists blinded to the study groups. Cleavage-stage embryos were evaluated on Day 3 according to the Istanbul Consensus criteria, focusing on blastomere number, fragmentation percentage, symmetry, and the presence of multinucleation. Grade A (excellent) was defined as 7–8 cells with <10% fragmentation and equal-sized blastomeres. For cases reaching the blastocyst stage, the Gardner classification was utilized, assessing the degree of expansion (1–6) and the quality of the inner cell mass and trophectoderm (A–C).

Sample size calculation was based on previous studies evaluating dual triggering in poor responders (Zhang *et al.* 2017). Assuming a minimum clinically significant difference of 1.5 MII oocytes between groups, a standard deviation of 3.0, 80% power, and a two-sided α of 0.05, 64 participants were required per group. To compensate for a projected dropout rate of 15–20%, the final sample size was increased to 76 women per group (total *n* = 152).

Eligible participants were women younger than 42 years with a body mass index (BMI) of 18–28 kg/m^2^, laparoscopically and pathologically confirmed endometriosis, and POR according to the ESHRE Bologna criteria. Participants were required to meet at least two of the following criteria: age ≥ 40 years, previous POR with ≤3 retrieved oocytes, or abnormal ovarian reserve testing (AFC < 5 and AMH 0.5–1.1 ng/mL). Additional eligibility criteria included an indication for ICSI. Exclusion criteria comprised diabetes mellitus, hyperprolactinemia, thyroid disorders, congenital adrenal hyperplasia, Cushing syndrome, polycystic ovary syndrome, uterine anomalies, recurrent implantation failure, day-3 FSH ≥ 15 IU/L, and azoospermia.

Participants were randomized using a computer-generated block randomization sequence with a block size of four, generated by an independent researcher not involved in clinical care. Allocation concealment was maintained with sequentially numbered, opaque, sealed envelopes. The study used a double-blind design. Patients were blinded to their treatment allocation through a placebo injection (2 mL of distilled water) in the control group. Clinicians performing the oocyte retrieval and embryologists, who served as the primary outcome assessors evaluating oocyte maturity, fertilization, and embryo quality, remained blinded to group assignments throughout the study. The statistical analyst also remained blinded to the treatment groups until the final analysis to prevent detection bias.

We initiated controlled ovarian stimulation on day 3 of the menstrual cycle following baseline ultrasonography. Recombinant FSH (Cinnal-F; CinnaGen, Iran) and human menopausal gonadotropin (HMG) were administered at an initial dose of 300 IU/day, adjusted according to age, antral follicle count, and baseline FSH level. Follicular response was assessed by transvaginal ultrasound on day 5, after which a GnRH antagonist (Cetronax) was initiated according to a fixed protocol. Serial ultrasound examinations were subsequently performed to monitor follicular growth.

When the leading follicle reached 18–20 mm, final oocyte maturation was triggered. The control group received 250 μg recombinant hCG (Ovitrelle 250 μg/0.5 mL) plus placebo, whereas the dual-trigger group received 250 μg recombinant hCG followed by 0.2 mg GnRH agonist (CinnaFact), with both administered subcutaneously.

Oocyte retrieval was performed 34 h after triggering under transvaginal ultrasound guidance. Fertilization was achieved using ICSI, and all embryos were cryopreserved. Frozen embryo transfer (FET) was subsequently performed using a hormone replacement therapy protocol. Estradiol valerate (6 mg/day) was initiated when endometrial thickness was <2–3 mm and continued for 10–12 days. Once endometrial thickness reached ≥6 mm with a trilaminar pattern, intramuscular progesterone (100 mg/day) was started and continued for five days before embryo transfer. Serum β-hCG levels were measured 14 days after embryo transfer, and values > 50 IU/mL were considered positive. Luteal phase support continued until the eighth week of pregnancy.

### Statistical analysis

All statistical analyses were conducted using IBM SPSS Statistics for Windows, version 22.0 (IBM Corp., USA). We assessed continuous variables for normality using the Shapiro–Wilk test and visual inspection of Q–Q plots. Categorical variables are presented as frequencies and percentages (*n*, %), whereas continuous variables are presented as mean ± standard deviation (SD). A two-tailed *P*-value <0.05 was established *a priori* as the threshold for statistical significance.

Analyses were conducted on two predefined populations:

**Per-protocol (PP) population**: comprised participants who successfully adhered to the allocated stimulation protocol, underwent oocyte retrieval, and proceeded to embryo transfer (*n* = 65 in the hCG group and *n* = 63 in the dual-trigger group). This analysis evaluates the true pharmacological and biological efficacy of the triggering regimens under optimal compliance conditions.

**Intention-to-treat (ITT) population**: encompassed all randomized participants who initiated the cycle (*n* = 76 per arm). To account for protocol deviations, cycle cancellations, and fertilization/embryo transfer failures without introducing attrition bias, we applied a conservative worst-case scenario analysis in which all dropouts and non-transferred cycles were treated as treatment failures. This approach provides a pragmatic estimate of clinical effectiveness in routine practice.

Baseline demographic and clinical characteristics were compared between study arms across the ITT cohort using Welch’s independent two-sample *t*-test, which does not assume equal variances, for normally distributed continuous variables. For skewed continuous and count parameters (e.g. baseline LH levels, gravidity, and previous live births), the nonparametric Mann–Whitney *U* test was employed.

For primary and secondary embryological and ovarian stimulation outcomes (PP population), we compared continuous parameters using independent-samples *t*-tests or Welch’s *t*-tests, as appropriate. Standardized mean differences (Cohen’s *d*) with corresponding 95% confidence intervals (95% CIs) were calculated to quantify effect sizes. Binary clinical outcomes (chemical pregnancy, clinical pregnancy, spontaneous abortion, and ectopic pregnancy) were evaluated using the Chi-square test or Fisher’s exact test when expected cell counts were <5. Binary reproductive outcomes are expressed as relative risks (RRs) with 95% CIs for both PP and ITT populations.

### Ethical considerations

This clinical trial was conducted after approval by the Ethics Committee of Tehran University of Medical Sciences (approval code: IR.TUMS.MEDICINE.REC.1400.1257) and registration in the Iranian Registry of Clinical Trials (IRCT20091012002576N22). The study procedures and the concept of assisted reproductive treatment were explained to both partners of each couple. Confidentiality of all personal and medical information was strictly maintained. Written informed consent was obtained from all participating couples prior to enrollment. Participants were also informed of their right to withdraw from the study at any time without affecting their medical care.

## Results

### Participant flow and recruitment

As shown in the CONSORT flow diagram (Fig. 1), 450 patients were initially screened for eligibility; 298 were excluded (212 did not meet the inclusion criteria and 86 declined to participate). The remaining 152 eligible participants were randomly assigned in a 1:1 ratio to either the rhCG group (*n* = 76) or the dual-trigger group (*n* = 76), constituting the intention-to-treat (ITT) population (*n* = 152). Following allocation, 11 patients in the rhCG group and 13 in the dual-trigger group experienced cycle cancellation due to poor ovarian response or fertilization/cleavage failure. Consequently, 128 patients (65 in the rhCG group and 63 in the dual-trigger group) successfully completed embryo transfer and comprised the per-protocol (PP) population.

**Figure 1:**
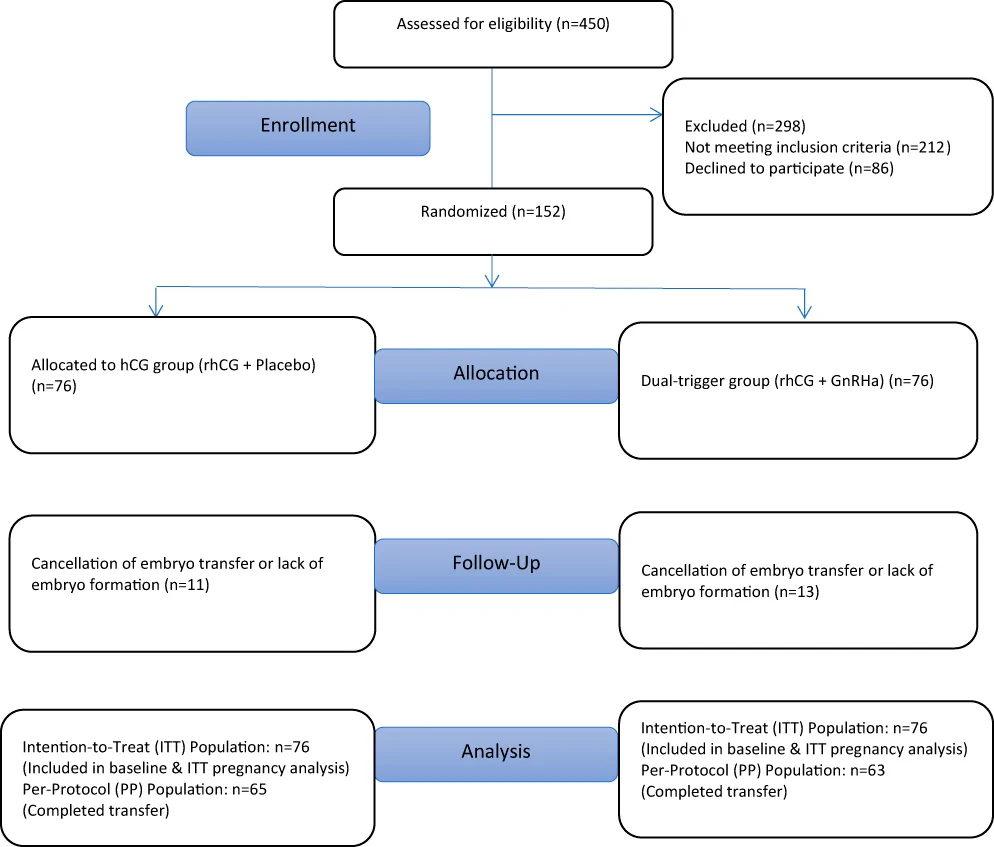
Flow chart of the clinical trial study.

### Baseline characteristics

Table 1 summarizes baseline demographic and clinical parameters for the ITT population (*n* = 76 per group). The two groups were comparable regarding maternal age (35.76 ± 3.88 vs 36.23 ± 4.32 years; *P* = 0.482), body mass index (25.55 ± 2.37 vs 26.06 ± 2.27 kg/m^2^; *P* = 0.178), gravidity (0.91 ± 1.01 vs 0.99 ± 1.07; *P* = 0.636), previous live births (0.41 ± 0.6 vs 0.26 ± 0.55; *P* = 0.134), history of intrauterine fetal death (0.05 ± 0.22 vs 0.03 ± 0.16; *P* = 0.523), history of ectopic pregnancy (0.04 ± 0.19 vs 0.13 ± 0.41; *P* = 0.085), baseline serum anti-Müllerian hormone (0.81 ± 0.26 vs 0.82 ± 0.25 ng/mL; *P* = 0.809), follicle-stimulating hormone (8.24 ± 4.41 vs 7.03 ± 4.05 IU/L; *P* = 0.080), estradiol (49.54 ± 31.39 vs 53.37 ± 37.59 pg/mL; *P* = 0.496), and thyroid-stimulating hormone levels (2.09 ± 1.11 vs 2.11 ± 1.17 mIU/L; *P* = 0.914). However, the hCG group had a longer duration of infertility (4.05 ± 3.13 vs 3.14 ± 2.49 years; *P* = 0.049), higher baseline luteinizing hormone levels (7.58 ± 10.30 vs 4.95 ± 2.01 IU/L; *P* = 0.032), and greater baseline endometrial thickness (8.04 ± 1.01 vs 7.61 ± 0.95 mm; *P* = 0.008) than the dual-trigger group (Table 1).

**Table 1:** Average distribution, standard deviation, and comparison of groups participating in the study by the general variables studied. Data are expressed as mean (SD). Analyses were performed on the intention-to-treat population (including all randomized patients (*n* = 76 per group)). *P*-values were calculated using Welch’s independent *t*-test; degrees of freedom ranged from 80.7 (LH) to 149.8 (AMH) via the Welch–Satterthwaite approximation. Due to the skewed distribution of LH, gravidity, and live births, these were analyzed using the Mann–Whitney *U* test; all other continuous variables were compared with Welch’s *t*-test.

	hCG group (*n* = 76)	Dual-trigger (*n* = 76)	*P*-value
Age (years)	35.76 (3.88)	36.23 (4.32)	0.482
BMI (kg/m^2^)	25.55 (2.37)	26.06 (2.27)	0.178
Gravid	0.91 (1.01)	0.99 (1.07)	0.636
Live births	0.41 (0.67)	0.26 (0.55)	0.134
IUFD	0.05 (0.22)	0.03 (0.16)	0.523
EP	0.04 (0.19)	0.13 (0.41)	0.085
Years of infertility	4.05 (3.13)	3.14 (2.49)	0.049
AMH (ng/mL)	0.81 (0.26)	0.82 (0.25)	0.809
LH (IU/L)	7.58 (10.30)	4.95 (2.01)	0.032
FSH (IU/L)	8.24 (4.41)	7.03 (4.05)	0.080
Estradiol (pg/mL)	49.54 (31.39)	53.37 (37.59)	0.496
TSH (mIU/L)	2.09 (1.11)	2.11 (1.17)	0.914
Endometrial thickening (mm)	8.04 (1.01)	7.61 (0.95)	0.008

BMI, body mass index; IUFD, intrauterine fetal death; EP, ectopic pregnancy; AMH, anti-Müllerian hormone; LH, luteinizing hormone; FSH, follicle-stimulating hormone; TSH, thyroid-stimulating hormone; SD, standard deviation.

### Ovarian stimulation and embryological outcomes

Table 2 presents the primary and embryological outcomes among patients who reached oocyte retrieval and embryo transfer (PP population: *n* = 65 in the hCG group vs *n* = 63 in the dual-trigger group). The mean number of retrieved oocytes was significantly higher in the dual-trigger arm than in the hCG arm (7.63 ± 4.84 vs 5.42 ± 3.52; effect size (ES) = 0.52, 95% CI: 0.19–0.84; *P* = 0.002). In evaluating the primary outcome, the dual-trigger regimen achieved a significantly higher yield of mature MII oocytes (6.53 ± 4.61 vs 4.14 ± 2.91; ES = 0.63, 95% CI: 0.30–0.95; *P* < 0.001). Retrieval of immature oocytes did not differ significantly between groups for MI (0.76 ± 0.97 vs 0.91 ± 0.96; ES = −0.21, 95% CI: −0.53 to 0.10; *P* = 0.141) or GV oocytes (0.46 ± 0.75 vs 0.53 ± 0.87; ES = −0.06, 95% CI: −0.38 to 0.25; *P* = 0.620). Regarding laboratory fertilization and embryo parameters, the dual-trigger group had significantly more successfully fertilized oocytes (5.07 ± 3.52 vs 3.19 ± 2.33; ES = 0.63, 95% CI: 0.30–0.96; *P* < 0.001) and frozen embryos (4.58 ± 3.93 vs 2.80 ± 2.35; ES = 0.55, 95% CI: 0.22–0.87; *P* = 0.001). While the mean number of transferred embryos was similar between the two groups (1.91 ± 1.02 vs 2.08 ± 0.98; ES = −0.17, 95% CI: −0.49 to 0.14; *P* = 0.291), embryo quality grading favored the dual-trigger protocol: the number of Grade A embryos was significantly higher (0.66 ± 0.82 vs 0.37 ± 0.62; ES = 0.39, 95% CI: 0.05–0.73; *P* = 0.023), whereas the number of Grade C embryos was markedly lower (0.10 ± 0.46 vs 0.84 ± 1.02; ES = −0.92, 95% CI: −1.27 to −0.56; *P* < 0.001). The number of Grade B embryos did not differ significantly between cohorts (0.63 ± 0.71 vs 0.85 ± 0.74; ES = −0.30, 95% CI: −0.63 to 0.03; *P* = 0.083). In addition, the mean number of successful implantations was significantly greater in the dual-trigger group (0.43 ± 0.58 vs 0.11 ± 0.31; ES = 0.68, 95% CI: 0.32–1.03; *P* < 0.001) (Table 2).

**Table 2:** Main results of ovarian stimulation by study protocol (primary outcome). Data are expressed as mean (SD). Analyses were performed on the per-protocol population (those who underwent embryo transfer).

	hCG group	Dual-trigger	Effect size (95% CI)	*P*-value
	(*n* = 65)	(*n* = 63)		
Retrieved oocytes	5.42 (3.52)	7.63 (4.84)	0.52 (0.19–0.84)	0.002
MI oocytes	0.91 (0.96)	0.76 (0.97)	−0.21 (−0.53 to 0.10)	0.141
MII oocytes	4.14 (2.91)	6.53 (4.61)	0.63 (0.30–0.95)	<0.001
GV oocytes	0.53 (0.87)	0.46 (0.75)	−0.06 (−0.38 to 0.25)	0.620
Frozen embryos	2.80 (2.35)	4.58 (3.93)	0.55 (0.22–0.87)	0.001
Number of transferred embryos	2.08 (0.98)	1.91 (1.02)	−0.17 (−0.49 to 0.14)	0.291
Grade A embryos	0.37 (0.62)	0.66 (0.82)	0.39 (0.05–0.73)	0.023
Grade B embryos	0.85 (0.74)	0.63 (0.71)	−0.30 (−0.63 to 0.03)	0.083
Grade C embryos	0.84 (1.02)	0.10 (0.46)	−0.92 (−1.27 to −0.56)	<0.001
Successful fertilization	3.19 (2.33)	5.07 (3.52)	0.63 (0.30–0.96)	<0.001
Successful implantations	0.11 (0.31)	0.43 (0.58)	0.68 (0.32–1.03)	<0.001

MI, metaphase I; MII, metaphase II; GV, germinal vesicle; SD, standard deviation; CI, confidence interval.

### Pregnancy outcomes (secondary outcomes)

Table 3 presents the reproductive outcomes, evaluated using both per-protocol (efficacy) and intention-to-treat (effectiveness) frameworks. In the per-protocol population (*n* = 65 hCG group vs *n* = 63 dual-trigger group), the chemical pregnancy rate was significantly higher in the dual-trigger group than in the hCG group (38.1% (24/63) vs 15.4% (10/65); relative risk of success (RR) = 2.48, 95% CI: 1.29–4.75; *P* = 0.004). In the ITT analysis (*n* = 76 per group, treating cycle cancellations as failures), the dual-trigger protocol maintained a statistically significant higher chemical pregnancy rate (31.6% (24/76) vs 13.2% (10/76); RR = 2.40, 95% CI: 1.23–4.67; *P* = 0.006).

**Table 3:** Main results of embryo implantation (secondary outcome). No imputation methods were applied, as all missing outcomes were classified as failures in the ITT analysis (worst-case scenario approach). Relative risk (RR) and 95% confidence intervals (CI) were calculated using the Wald approximation method. *P*-values were determined using the chi-square test or Fisher’s exact test, where appropriate.

	Drug group, *n* (%)		RR (95% CI)	*P*-value
	hCG group	Dual-trigger		
Positive chemical pregnancy				
PP*	10 (15.4)	24 (38.1)	2.48 (1.29–4.75)	0.004
ITT^†^	10 (13.2)	24 (31.6)	2.40 (1.23–4.67)	0.006
Positive clinical pregnancy				
PP	7 (10.8)	24 (38.1)	3.54 (1.64–7.62)	<0.001
ITT	7 (9.2)	24 (31.6)	3.43 (1.57–7.48)	<0.001
Abortion				
PP	4 (6.2)	10 (15.9)	2.58 (0.85–7.80)	0.078
ITT	4 (5.3)	10 (13.2)	2.50 (0.82–7.62)	0.092
EP				
PP	0 (0)	2 (3.2)	5.16 (0.25–105.34)	0.240
ITT	0 (0)	2 (2.6)	5.00 (0.24–102.44)	0.497

EP, ectopic pregnancy; RR, relative risk for success; CI, confidence interval.

* The per-protocol (PP) population comprised patients who completed the study protocol (*n* = 65 in the hCG group and *n* = 63 in the dual-trigger group), providing an estimate of treatment efficacy.

^†^ The intention-to-treat (ITT) population included all randomized patients (*n* = 76 per group), with dropouts and cycle cancellations classified as treatment failures, thereby minimizing attrition bias and providing a pragmatic estimate of treatment effectiveness.

Similarly, clinical pregnancy rates were significantly higher with dual triggering across both analytical sets: in the PP cohort, clinical pregnancy was achieved in 38.1% (24/63) of patients in the dual-trigger group compared with 10.8% (7/65) in the hCG group (RR = 3.54, 95% CI: 1.64–7.62; *P* < 0.001). Under ITT conditions, clinical pregnancy was observed in 31.6% (24/76) vs 9.2% (7/76), respectively (RR = 3.43, 95% CI: 1.57–7.48; *P* < 0.001).

The rate of spontaneous abortion did not differ significantly between the dual-trigger and hCG groups in either the PP analysis (15.9% (10/63) vs6.2% (4/65); RR = 2.58, 95% CI: 0.85–7.80; *P* = 0.078) or the ITT analysis (13.2% (10/76) vs 5.3% (4/76); RR = 2.50, 95% CI: 0.82–7.62; *P* = 0.092). Ectopic pregnancy occurred in two patients in the dual-trigger group (3.2% in PP, 2.6% in ITT) and in no patients in the hCG group, with no statistically significant difference between arms (PP: RR = 5.16, 95% CI: 0.25–105.34; *P* = 0.240; ITT: RR = 5.00, 95% CI: 0.24–102.44; *P* = 0.497) (Table 3).

## Discussion

In this single-center randomized clinical trial with a limited sample size, the mean number of MII oocytes was significantly greater in patients receiving dual triggering. In addition, both chemical and clinical pregnancy rates were significantly higher in the dual-trigger group compared with the hCG-only group. The dual-trigger group also demonstrated a significantly higher mean number of Grade A embryos than the hCG group. These findings suggest that dual triggering may improve oocyte maturation and embryo quality, resulting in higher-quality embryos than those obtained using hCG alone. Furthermore, certain confounding factors warrant consideration. Although all participants were categorized as poor responders, the clinical heterogeneity (including the stage of endometriosis and prior surgical interventions) could potentially influence the response to the dual-trigger protocol. Although randomization was used to distribute these variables evenly between groups, future studies with larger cohorts should consider stratified analysis by endometriosis severity (stages I–IV) to better clarify the differential impact of triggering strategies.

Four meta-analyses indicate that endometriosis, particularly in the presence of ovarian endometriomas, is associated with fewer retrieved mature oocytes and, in many cases, reduced fertilization rates, suggesting a detrimental impact on oocyte quality. However, interpretation of these findings is challenging due to several confounding factors, including disease stage, prior medical or surgical treatment, and considerable heterogeneity across studies (Barnhart *et al.* 2002, Harb *et al.* 2013, Yang *et al.* 2015, Rossi & Prefumo 2016). Evidence from oocyte donation programs further supports the hypothesis that the primary impairment is related to the oocyte rather than the uterine environment, as recipients with endometriosis achieve comparable reproductive outcomes when using oocytes from healthy donors. In clinical practice, ICSI is often preferred over conventional IVF in these patients to enhance fertilization outcomes (Sanchez *et al.* 2017). Conversely, the potential benefits of pretreatment with GnRH agonists or surgical intervention to improve oocyte quality remain uncertain, and the role of fertility preservation strategies using cryopreserved oocytes in women with endometriosis has not yet been fully elucidated (Somigliana *et al.* 2015).

Several studies have evaluated the potential benefits of dual triggering on oocyte maturation and embryo quality. In a clinical trial conducted by Haas *et al.* (2019) among poor-responder women, the mean number of high-quality embryos in the dual-trigger group (1.1 ± 0.9) was significantly higher than in the comparison groups (0.5 ± 0.7 and 0.3 ± 0.8; *P* < 0.02). Similarly, Decleer *et al.* (2014) reported that the combination of GnRH-a with 5,000 IU of hCG resulted in a greater proportion of patients with at least one high-quality embryo and a higher number of frozen embryos, although no significant differences were observed in the mean number of mature MII oocytes or pregnancy rates. Ali *et al.* (2020) demonstrated that among normal-responder women, dual triggering was associated with a significantly higher number of retrieved oocytes and improved embryo quality; however, fertilization rate, implantation rate, clinical pregnancy, and live birth outcomes were comparable between the groups. These findings suggest that the combined administration of GnRH-a and r-hCG may enhance oocyte maturation and embryo developmental potential in women undergoing ICSI. Consistent with these observations, Lin *et al.* (2013) reported significantly higher rates of mature oocytes, implantation, and clinical pregnancy in the dual-trigger group. In addition, a review by Orvieto (2015) examining studies on the use of GnRH-a together with hCG for final follicular maturation concluded that, in patients not at risk for ovarian hyperstimulation syndrome, the combined administration of GnRH-a and hCG yields oocyte and embryo quality comparable to or superior to that achieved with hCG alone. The authors further suggested that dual triggering may enhance ART outcomes when GnRH-a and hCG are administered simultaneously, approximately 34–37 h before oocyte retrieval in normal or poor responders and 34–40 h before retrieval in patients with abnormal oocyte maturation.

Some studies have reported findings that contrast with the results of the present study. Shakerian *et al.* (2021) evaluated 50 IVF/ICSI candidates who were not at risk of ovarian hyperstimulation syndrome (OHSS) and found no significant differences between the hCG and dual-trigger groups in terms of the number of retrieved oocytes or pregnancy outcomes. Their findings suggested that dual triggering did not improve ICSI outcomes. Similarly, in a randomized trial involving 192 normal responders, Eftekhar *et al.* (2017) reported significantly higher numbers of retrieved oocytes and embryos in the dual-trigger group; however, pregnancy and implantation rates were comparable between the groups, indicating that dual triggering did not significantly improve overall pregnancy outcomes. In addition, Griffin *et al.* (2014) investigated women with more than 25% immature oocyte retrieval and observed that although dual triggering increased the proportion of mature oocytes, overall IVF outcomes remained suboptimal in both groups.

The strengths of the present study include its double-blind randomized design, clearly defined inclusion and exclusion criteria, and relatively homogeneous study groups. Furthermore, multiple clinically relevant primary and secondary outcomes were evaluated, providing a comprehensive assessment of treatment effects. However, several limitations should also be acknowledged. The main limitations include the relatively small sample size and the single-center design, which were largely influenced by time constraints and limited financial resources. Another limitation of our study is the lack of live birth rate (LBR) data, which is the ultimate goal of IVF/ICSI treatments. Future prospective trials are warranted to evaluate whether the improvements in oocyte quality and clinical pregnancy rates translate into higher LBRs in this patient population.

The findings of this study suggest that the use of a dual-trigger protocol combining hCG and a GnRH-a was associated with a higher number of MII oocytes and an increased number of Grade A embryos. In addition, dual triggering may improve both chemical and clinical pregnancy rates in women with POR and endometriosis undergoing ICSI. These results indicate that individualized triggering strategies based on patient characteristics and ovarian response may help optimize ART outcomes in this specific population.

## Conclusion

In conclusion, this single-center randomized clinical trial with a limited sample size suggests that dual triggering with a GnRH agonist plus recombinant hCG increases the number of mature oocytes and improves pregnancy outcomes compared with recombinant hCG alone. Further large-scale, multicenter trials are warranted to confirm these findings.

## Declaration of interest

The authors declare that there is no conflict of interest that could be perceived as prejudicing the impartiality of the work reported.

## Funding

This research received no specific grant from any funding agency in the public, commercial, or not-for-profit sector.

## Author contribution statement

 FDT and ME conceptualized the study objectives and methodology. ER, SZ and EF curated the data and performed formal analyses. ER and ZK drafted the initial manuscript. HR and SR critically reviewed and edited the manuscript. All authors contributed to the study design, analysis, interpretation of the findings, and final approval of the manuscript.

## Data availability

The data supporting the findings of this study are available from the corresponding author upon reasonable request. Access will require a signed data-sharing agreement and approval by an independent review committee. The request may include de-identified individual participant data underlying the results reported in this article, as well as supporting materials such as tables, figures, appendices, and the study protocol.

## Ethical statement

The clinical trial received approval from the Ethics Committee of Tehran University of Medical Sciences under the code IR.TUMS.MEDICINE.REC.1400.1257 (approval date: 2022-01-29).

## RCT registration

The clinical trial was registered in the Iranian Registry of Clinical Trials with the code IRCT20091012002576N22. Site: https://irct.behdasht.gov.ir/trial/61711 (registration date: 2022-02-15; initial participant enrollment date: 2022-04-01).

## Declaration of AI and AI-assisted technologies in the writing process

The authors used Grammarly AI software and ChatGPT 5.6 to improve readability, grammar, and text integrity. The authors supervised and approved all content.
